# Mechanistic Pathways Underlying the Antihypertensive Effect of Fermented Milk with *Lactococcus lactis* NRRL B-50571 in Spontaneously Hypertensive Rats

**DOI:** 10.3390/nu10030262

**Published:** 2018-02-26

**Authors:** Lilia M. Beltrán-Barrientos, Adrián Hernández-Mendoza, Aarón F. González-Córdova, Humberto Astiazarán-García, Julián Esparza-Romero, Belinda Vallejo-Córdoba

**Affiliations:** Centro de Investigación en Alimentación y Desarrollo, A.C. (CIAD), Carretera a La Victoria Km. 0.6, Apartado 1735, Hermosillo, Sonora 83304, Mexico; lilia.beltranb@gmail.com (L.M.B.-B.); ahernandez@ciad.mx (A.H.-M.); aaronglz@ciad.mx (A.F.G.-C.); hastiazaran@ciad.mx (H.A.-G.); julian@ciad.mx (J.E.-R.)

**Keywords:** fermented milk, *Lactococcus lactis*, angiotensin converting enzyme inhibition, nitric oxide, antioxidant activity, opioid effect

## Abstract

It has been reported that fermented milk (FM) with *Lactococcus lactis* NRRL B-50571 had an antihypertensive effect in spontaneously hypertensive rats (SHR) and prehypertensive subjects. Therefore, the objective of the present study was to evaluate the possible mechanisms involved (angiotensin converting enzyme inhibition (ACEI), enhancement of nitric oxide production, antioxidant activity and opioid effect), in the antihypertensive effect of FM with SHR. First, twenty one SHR were randomized into three groups to either receive in a single-oral dose of purified water (negative control), FM, or naloxone (opioid receptor antagonist) + FM. In a parallel study, twenty seven SHR were randomized into three groups to either receive ad libitum purified water (negative control), Captopril or FM. After six weeks of treatment ACEI activity, enhancement of nitric oxide production, and antioxidant activity were evaluated in plasma. Results indicated that opioid receptors were not involved in the hypotensive effect of FM. However, ACEI activity (94 U/L), the oxidative stress index (malondialdehyde/catalase + glutathione peroxidase) 0.9, and nitric oxide in plasma (4.4 ± 1.3 U/L), were significantly different from the negative control, and not significantly different from the Captopril group. Thus, these results suggested that these mechanisms are involved in the hypotensive effect of FM.

## 1. Introduction

Hypertension is an important risk factor for cardiovascular diseases, a leading risk factor for death and disability. It has been estimated that hypertension affects more than 40% of people over 25 [1]. The high cost and adverse effects associated with pharmacological therapy have encouraged the scientists to search for new alternatives [2]. Therefore, there has been a rising interest in fermented dairy foods that, besides being nutritional, may promote health or reduce diseases, such as hypertension [3,4]. The beneficial effects of dairy products are attributed to several bioactive components, such as calcium, medium-chain fatty acids, lactose, conjugated linoleic acid and bioactive peptides [5]. Bioactive peptides from milk are liberated from the native protein through proteolysis during gastrointestinal digestion or food processing, such as fermentation with lactic acid bacteria (LAB) [6]. In fact, multifunctional properties of milk-derived peptides are increasingly recognized [7]. 

The antihypertensive effect of fermented milk products has been attributed to bioactive peptides [8] and/or gamma-amminobutyric acid (GABA) [9] produced during milk fermentation. In fact, the antihypertensive effect of bioactive peptides is often attributed to angiotensin-I converting enzyme inhibition (ACEI), an enzyme that plays a crucial role in blood regulation through the renin angiotensin system (RAS) [10]. Nevertheless, it has been reported that in some cases there is no correlation between in vitro and in vivo ACEI activity, due to peptides undergoing further degradation during gastrointestinal digestion, which may cause less bioavailability to reach target organs and cause the beneficial effect [8]. However, peptides with antioxidant [11], nitric oxide pathway [12], and opioid receptor binding activities [13] might also exhibit antihypertensive activity. Hence, antihypertensive bioactive peptides in fermented milks, may be acting via multiple mechanisms [8].

It has been previously reported that a fermented skim milk product with *Lactococcus* (*L.*) *lactis* NRRL B-50571 had ACEI activity in vitro; and this effect was strain-dependent [14,15]. Furthermore, fermented milk with *L. lactis* NRRL B-50571 reduced systolic blood pressure (SBP) and diastolic blood pressure (DBP), heart rate and had a hypolypidemic effect on spontaneously hypertensive rats (SHR) [16,17]. Additionally, in a pilot randomized double blind controlled clinical trial with prehypertensive subjects a blood pressure lowering effect of fermented milk with *L. lactis* NRRL-B50571 was observed [18]. Afterwards, we assessed that the antihypertensive effect of fermented milk with *L. lactis* was not due to the GABA present when it was administered to SHR [19]. Hence, the antihypertensive effect may be attributed to bioactive peptides present in this fermented milk; yet, it is not clear which mechanism is involved in the hypotensive effect. Therefore, the aim of the present study was to determine in SHR if the antihypertensive effect of fermented milk with *L. lactis* NRRL B-50571 was through the nitric oxide pathway, the opioid receptor binding, or the ACEI and antioxidant activities.

## 2. Materials and Methods

### 2.1. Strains and Growth Conditions

*L. lactis* strain NRRL B-50571 was propagated as previously reported by Rodríguez-Figueroa et al. [14] in 10 mL of sterile lactose (10%, *w*/*v*) M17 broth (DIFCO, Sparks, MD) and incubated at 30 °C for 24 h. Fresh precultures were obtained by repeating the same procedure twice to allow growth until reaching 10^6^ to 10^7^ cfu/mL. To obtain a working culture, a fresh culture was inoculated (3%) in sterile (110 °C, 10 min) nonfat dry milk reconstituted (10%, *w*/*w*) and incubated at 30 °C for 12 h.

### 2.2. Sample Preparation

Fermented milk with *L. Lactis* NRRL B-50571 (FM) was prepared as previously reported [18]. Reconstituted (10%, *w*/*v*) commercial skim milk was pasteurized (80 °C for 30 min), inoculated with 3% working culture and fermented at 30 °C for 48 h. To inactivate LAB, fermentation was stopped by applying heat treatment (75 °C, 15 min), followed by quick cooling; subsequently fermented milk was frozen (–20 °C), for further analysis.

To obtain the lyophilized water-soluble extracts from *L. lactis* fermented milk with NRRL B-50571 (WSE-FM) for the evaluation of the opioid effect, WSE-FM were obtained by centrifugation (ThermoScientific, Chelmsford, MA, USA) at 5000 rpm for 40 min at 4 °C; then lyophilized with a freeze-dryer (Labconco, Kansas City, MO, USA), and kept at 4 °C until use for further analysis. Total protein content (Method 960.52 AOAC, 1998) of the lyophilized extracts was evaluated.

### 2.3. In Vivo Experimental Protocols

A total of twenty-nine male SHR (4 weeks old; 44.7 ± 5.15 g body weight (BW)) were obtained from Charles River Laboratories International, Inc. (Wilmington, MA, USA). Rats were housed in individual cages at 21 ± 2 °C, 12 h light–dark cycles and 52 ± 6% relative humidity, with an ad libitum intake of a standard diet (Purina, Cd. México, México) and purified water. Blood pressure was monitored every week until all rats developed hypertension according to Okamoto and Aoki [20]. SBP and DBP were taken 3 times using the non-invasive blood pressure system using a photoelectric sensor, amplifier, manual inflation cuff and software (Model 229; IITC Life Science Inc., (Woodland Hills, CA, USA). Once all rats were hypertensive, the possible antihypertensive mechanisms (opioid, ACEI, antioxidant, and nitric oxide pathway) were evaluated. All procedures involving animals were approved by the Bioethics Committee of the Research Center for Food and Development (Spanish acronym, CIAD), Hermosillo, Sonora, Mexico, (CE/009/2015).

### 2.4. Evaluation of Opioid Effect

When SHR were 16 weeks old (320.8 ± 16 g BW, 187.6 ± 15.6 mmHg SBP and 129.6 ± 16.9 mmHg DBP); twenty-one SHR were randomized into three groups (Table 1) of seven rats (*n* = 7). Treatments were assigned randomly to each group to either receive in a single dose: purified water (negative control); 35 mg protein of WSE-FM/kg animal BW; or 1 mg/kg animal BW of naloxone (μ-opioid antagonist receptor) (PiSa Farmacéutica, Cd. México, México) + 35 mg protein of FM-WSE/kg animal BW. FM-WSE from fermented milk was dissolved in purified water. 

In order to prepare the corresponding dose of naloxone and WSE-FM, the SHR were weighed before administration. Conscious SHR received via subcutaneous (s.c.) naloxone, and afterwards a single oral dose of WSE-FM through a gastric cannula between 8:00 and 10:00 hours to eliminate circadian cycles. SBP and DBP were monitored before treatment administration and every 10 min until 60 min post-administration.

### 2.5. Long-Term Effect of FM on Blood Pressure

Twenty-seven male SHR (19 weeks old, 346.4 ± 17.7 g BW, 201.5 ± 15.4 mmHg SBP and 153.4 ± 24.6 mmHg DBP), were randomized into three groups (Table 2) of nine rats (*n* = 9). SHR from the first study had a three-week washout period, before group allocation; during this time, blood pressure was monitored to assess any residual effect. Treatments were assigned randomly to each group to either receive: purified water (negative control); Captopril (a proven hypotensive drug, 40 mg/kg of BW, Sigma-Aldrich Co., St. Louis, MO, USA); or FM. All SHR had free access (ad libitum) to each treatment during the 6 weeks as part of the protocol. SBP and DBP were measured 3 times, once a week between 8:00 and 10:00 hours to eliminate circadian cycles. After six weeks of treatment, rats were sacrificed and blood samples were collected to immediately obtain plasma, and freezed −80 °C for further analysis.

### 2.6. ACEI Activity in Plasma

Plasma was used for measuring the ACEI activity according to the method of Cushman and Cheung [21]. Hippuryl-l-histidyl-l-leucine (a substrate for ACE) (Sigma-Aldrich Co., St. Louis, MO, USA) was dissolved in 0.1 mol/L sodium borate buffer (pH 8.3) containing 0.3 mol/L NaCl. The reaction was initiated by the addition of 100 μL of the serum and vascular tissue enzyme extract, then incubated for 30 min at 37 °C and stopped by the addition of 250 μL of 1 mol/L HCl. The hippuric acid liberated by ACE was extracted with 1.5 mL ethyl acetate, dissolved by addition of 1.0 mL of Milli-Q water, after removal of ethyl acetate by heating for 20 min at 75 °C, and measured at 228 nm. One unit (U) of activity was defined as the amount of enzyme, which released 1.0 mmol of hippuric acid/min under the above conditions. The specific activity of ACE in serum is expressed as U/L.

### 2.7. Nitric Oxide Pathway

Nitric oxide pathway was estimated through nitric oxide (NO) production. Plasma NO concentration was determined by the Griess reaction with a colormetric assay kit (Cell Biolabs, Inc., San Diego, CA, USA). This assay is based on the conversion of nitrate to nitrite by nitrate reductase, followed by quantification of nitrate after the Griess reaction. NO concentration in plasma was expressed as μmol/L.

### 2.8. Antioxidant Activity

Antioxidant activity from peptides was assessed indirectly by determining oxidative stress index/ratio. This index indicates the balance between lipoperoxidation (as malondialdehyde, MDA) and total antioxidant enzyme activity (catalase, CAT, and glutathione peroxidase, GPx) [22].

#### 2.8.1. Superoxide Dismutase Activity (SOD)

The determination of SOD activity in plasma was determined as described by Superoxide Dismutase assay kit (Cayman Chemical Company, Ann Arbor, MI, USA). In this assay, it determines the ability to inhibit the reduction of tetrazolium salt induced by xanthine-xanthine oxidase. One unit of SOD is defined as the amount of enzyme needed to exhibit 50% dismutation of the superoxide radical; and was expressed as U/mL.

#### 2.8.2. Catalase Activity Determination (CAT)

CAT activity in plasma determination was based on the decomposition of hydrogen peroxide (30 μM) at 240 nm [23]. CAT activity was defined as the amount of enzyme that removed 1 µmol H_2_O_2_ in 1 min. CAT activity was expressed as µmol H_2_O_2_/min/L.

#### 2.8.3. Glutathione Peroxidase (GPx) Activity

GPx activity in plasma was evaluated as described as the manufacturer for Glutathione peroxidase assay kit (Cayman Chemical Company, Ann Arbor, MI, USA). The GPx activity was determined 340 nm and is expressed as nmol/min/mL. 

#### 2.8.4. Determination of Lipid Peroxidation

The degree of lipid peroxidation in plasma was determined as described by Todorova et al. [24], with some modifications. This method uses thiobarbituric acid (TBA), which measures malondialdehyde (MDA) reactive products. 200 µL of plasma were mixed with 200 µL of PBS, and 200 µL trichloroacetic acid (25%). Afterwards, mixtures were centrifuged (2000 g, 20 min) and supernatants were mixed with 150 µL of TBA (1%), followed by heated at 95 °C for 1 h. After cooling absorbance was determined at 532 nm. The MDA concentration was calculated by using an extinction coefficient of 155 (1/mM·cm). MDA was expressed as µmol/L.

### 2.9. Statistical Analysis

Baseline systolic and diastolic blood pressures were defined as the mean of the values measured in the first run-in period. The blood pressure outcomes were presented as the mean value with standard deviations (SD) for all SHR in each group. For the evaluation of opioid effect the blood pressure (min 50 and 60 post-treatment) between groups were analyzed with one-way ANOVA. Differences among means were assessed by Scheffe multiple comparison test and considered significant when *p* < 0.05. For the long-term effect of FM on blood pressure, the outcomes for each week between groups were analyzed with one-way ANOVA. Differences among means were assessed by Scheffe multiple comparison test and considered significant when *p* < 0.05. The outcomes for ACEI activity, superoxide dismutase, GPx activity, MDA, and oxidative stress index, were analyzed with Kruskal–Wallis test since they did not presented a normal distribution; data are presented as medians and were considered significant when *p* < 0.05. The outcomes for nitric oxide and CAT activity were analyzed with one-way ANOVA. Differences among means were assessed by Scheffe multiple comparison test and considered significant when *p* < 0.05. Data are presented as means ± SEM.

## 3. Results and Discussion

The use of *Lactococcus lactis* NRRL B-50571 as a starter for fermented milk with antihypertensive effect has been reported in SHR [16,17] and prehypertensive subjects [18], and this hypotensive effect has been attributed to ACEI. Moreover, 21 identified peptides in this fermented milk possessed ACEI activity [15]. However, in several studies it has been reported that there is a lack of correlation between the in vitro and in vivo ACEI activity. This may be due to peptide degradation throughout gastrointestinal digestion, and this may implicate difficulty to reach the target organs in a sufficient amount to exert ACEI effect. However, it should not be disregarded that other mechanisms may be involved in the antihypertensive effect mediated through the interaction with receptor located at the gut [25]. Hence, to the best of our knowledge this is the first study that evaluates the possible mechanisms where peptides may be involved in the hypotensive effect; such as opioid, ACEI, antioxidant and NO pathway (by NO production). 

### 3.1. Opioid Effect

Twenty one SHR with blood pressure higher than 186/126 mmHg for systolic and diastolic blood pressures were eligible for randomization (Table 1). As expected, there were no significant differences (*p* > 0.05) between groups on clinical characteristics (systolic blood pressure, diastolic blood pressure, heart rate and weight).

**Figure 1:**
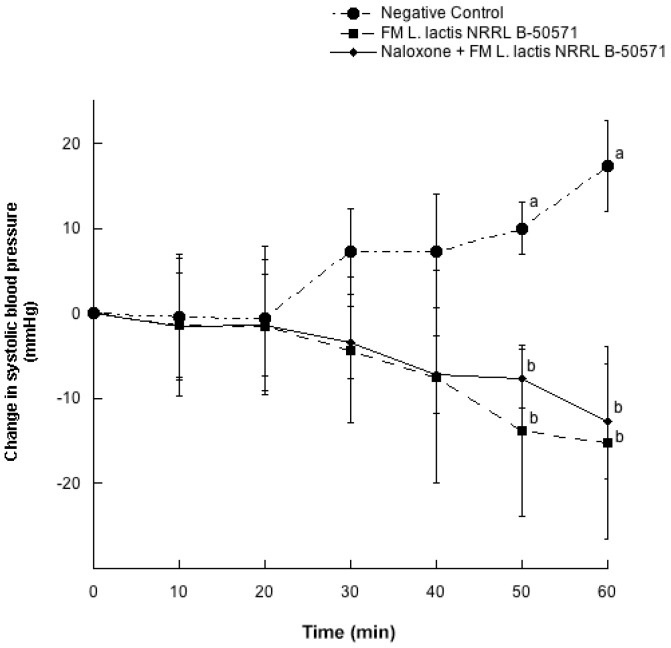
Change in systolic blood pressure in spontaneously hypertensive rats with different treatments. Negative control: purified water; FM *L. lactis* NRRL B-50571: lyophilized water-soluble extract (35 mg protein/kg body weight) of fermented milk with *Lactococcus lactis* NRRL B-50571; Naloxone: (1 mg/kg body weight) Antagonist μ opioid receptor + FM *L. lactis* NRRL B-50571: lyophilized water-soluble extract (35 mg protein/kg body weight) of fermented milk with *Lactococcus lactis* NRRL B-50571. Data are presented as means ± SEM. Data points sharing the same letter within a week was not significantly different (*p* > 0.05).

**Figure 2:**
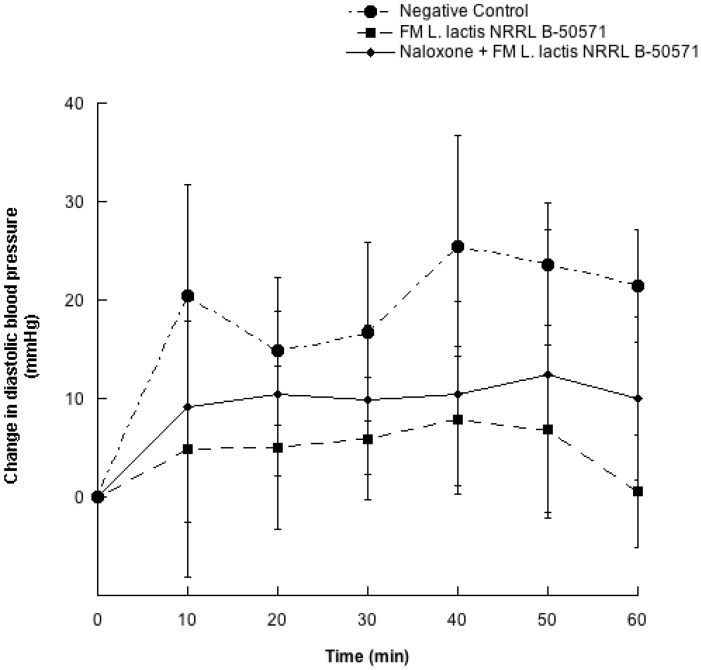
Change in diastolic blood pressure in spontaneously hypertensive rats with different treatments. Negative control: purified water; FM *L. lactis* NRRL B-50571: lyophilized water-soluble extract (35 mg protein/kg body weight) of fermented milk with *L. lactis* NRRL B-50571; Naloxone: (1 mg/kg body weight) Antagonist μ opioid receptor + FM *L. lactis* NRRL B-50571: lyophilized water-soluble extract (35 mg protein/kg body weight) of fermented milk with *L. lactis* NRRL B-50571. Data are presented as means ± SEM.

Figure 1 and Figure 2 depicts the changes of SBP and DBP every 10 min for 60 min after a single oral dose of purified water (negative control); WSE-FM; or naloxone + WSE-FM. After 50 and 60 min post-treatments, reductions on SBP from WSE-FM group and naloxone + WSE-FM group were 13.8 ± 26.1 and 7.7 ± 9.3 mmHg; and 15.2 ± 29.8 and 12.7 ± 17.7 mmHg, respectively; and were significantly different (*p* < 0.05) from the negative control. Though, DBP were not significantly different (*p* > 0.05) between all groups, DBP from groups receiving WSE-FM or naloxone + WSE-FM, tended to be slightly lower.

It has been previously reported that milk derived peptides possess opioid-like effect, and that this effect may exert a hypotensive effect through binding a specific μ opioid receptor [13]. In fact, the common structural feature for opioid milk peptides is the presence of tyrosine at the N-terminal end, and the presence of another aromatic residue [26]. Meanwhile, it was previously reported that three peptides with tyrosine at the N-terminal (YPSYGL, YPSYG and YIPIQYVLS) where present in the fermented milk with *L. lactis* NRRL B-50571 [15], therefore, we evaluated if the antihypertensive effect may be due to milk peptides binding to opioid receptors. 

In the present study μ-opioid receptors were blocked with antagonist opioid receptor (naloxone), and blood pressure reduction was not significantly different (*p* > 0.05) in WSE-FM group and naloxone + WSE-FM group. Hence, in this study the blood pressure lowering effect may not be attributed to peptides binding to opioid receptors.

On the other hand, it was reported that the mechanism of the blood pressure lowering effect of a tetrapeptide from milk whey, after a single subcutaneous administration to SHR, was by opioid receptors, since the response was antagonized with naloxone [13]. Thus, it should not be disregarded that if SHR were administered subcutaneously with specific peptides present in the fermented milk with *L. lactis* NRRL B-50571) [15], the antihypertensive effect could be via binding opioid receptors.

### 3.2. Long-Term Effect of FM on Blood Pressure

**Figure 3:**
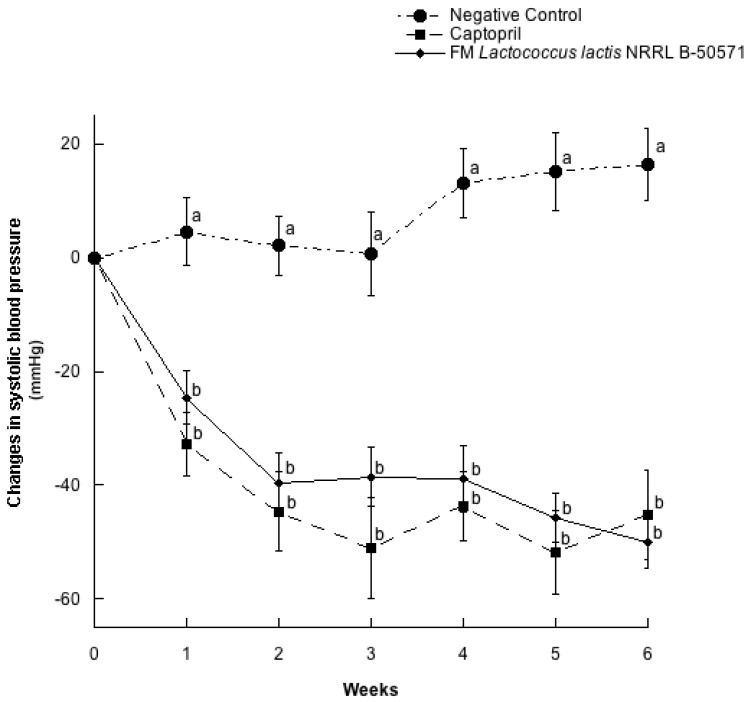
Change in systolic blood pressure in spontaneously hypertensive rats with different treatments. Negative control: purified water; Captopril: 40 mg/kg body weight; FM *L. lactis* NRRL B-50571 (ad libitum): fermented milk with *L. lactis* NRRL B-50571. Data are presented as means ± SEM. Data points sharing the same letter within a week was not significantly different (*p* > 0.05).

**Figure 4:**
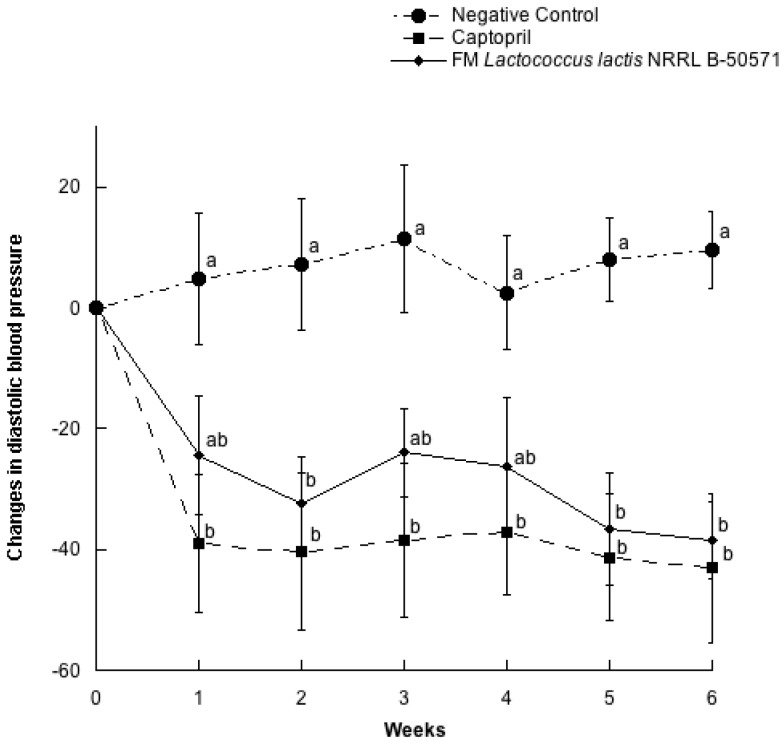
Change in diastolic blood pressure in spontaneously hypertensive rats with different treatments. Negative control: purified water; Captopril: 40 mg/kg body weight; FM *L. lactis* NRRL B-50571 (ad libitum): fermented milk with *L. lactis* NRRL B-50571. Data are presented as means ± SEM. Data points sharing the same letter within a week was not significantly different (*p* > 0.05).

In a parallel study, twenty seven SHR with blood pressure higher than 200/150 mmHg for systolic and diastolic blood pressures were selected for randomization (Table 2). As expected, there were no significant differences (*p* > 0.05) between groups on clinical characteristics (systolic blood pressure, diastolic blood pressure, heart rate and weight). In this study, we evaluated the other possible mechanisms involved in the antihypertensive effect of fermented milk with *L. lactis*, after six weeks of administration. Changes in SBP and DBP for every week are represented in Figure 3 and Figure 4. Both SBP and DBP values from the Captopril group and FM group were significantly different from the negative control group (*p* > 0.05), but they were not significantly different (*p* < 0.05) between them. Maximal SBP decreases from the FM group was of 49.9 ± 14.2 mmHg, after 6 weeks of treatment, while the Captopril group SBP decreased 45.2 ± 23.6 mmHg on the same week of intervention. After six weeks of intervention, SHR were sacrificed to obtain plasma and evaluate ACEI activity, NO production and antioxidant effect.

### 3.3. ACEI Activity

**Figure 5:**
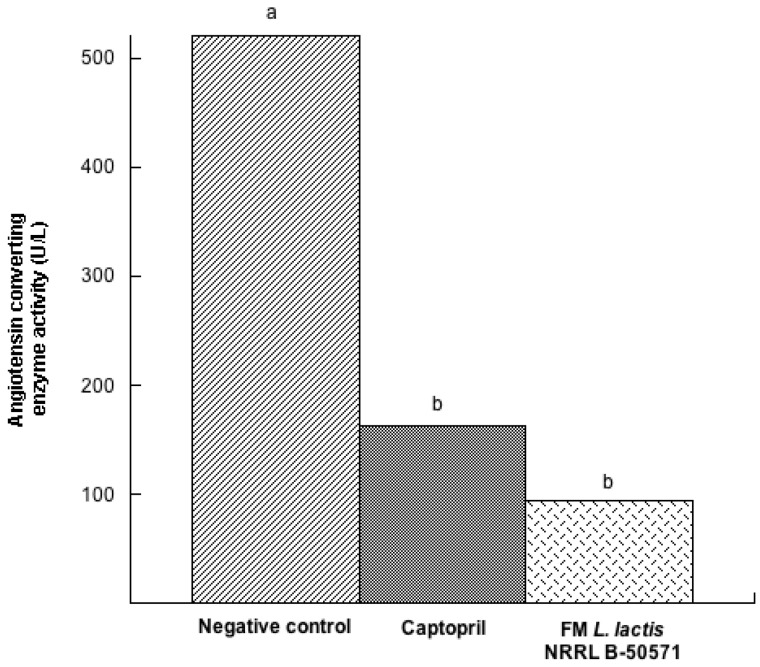
Angiotensin converting enzyme activity (U/L) in plasma from spontaneously hypertensive rats after long-term treatments. Negative control: purified water; Captopril: 40 mg/kg body weight; FM *L. lactis* NRRL B-50571 (ad libitum): fermented milk with *L. lactis* NRRL B-50571. Data are presented as median; and was analyzed by non-parametric test (Kruskal–Wallis *p* < 0.05). Data sharing the same letter was not significantly different (*p* > 0.05).

One method to determine *in vivo* ACEI activity indirectly from peptides is through the determination of circulating ACEI activity in plasma [27]. Figure 5 represents the ACEI activity in plasma of SHR after six weeks post-treatment. Plasma ACEI activity was reduced in SHR treated with Captopril (a potent ACE inhibitor) and FM groups, and was not significantly different between them (*p* > 0.05). Moreover, ACEI activity in plasma from the negative control group was 5.5 times higher than the FM group, and was significantly different (*p* < 0.05) from the groups treated with FM or Captopril. Thus, the effect may be attributed to the in vivo ACE inhibition from bioactive peptides in FM, because circulating ACE activity was reduced. 

A similar finding of ACEI activity in vivo after long-term effect of lactoferrin hydrolysates in SHR was evaluated [28]. In another study where there was a single intake of lactoferrin-derived peptides, there were also reductions in circulating ACE activity (40%) after 1 h post-administration; furthermore, these effects were similar to the SHR group, which received Captopril [29], henceforth angiotensin converting enzyme is an enzyme that plays a crucial role in blood regulation through the renin angiotensin system (RAS), thus its inhibition exerts antihypertensive effects [30]. In the present study, since reductions of the ACEI activity in circulating plasma were similar in the FM group and Captopril group; henceforth we may assume that this mechanistic pathway may be involved in the hypotensive effect of FM with *L. lactis* NRRL B-50571.

### 3.4. NO Pathway

**Figure 6:**
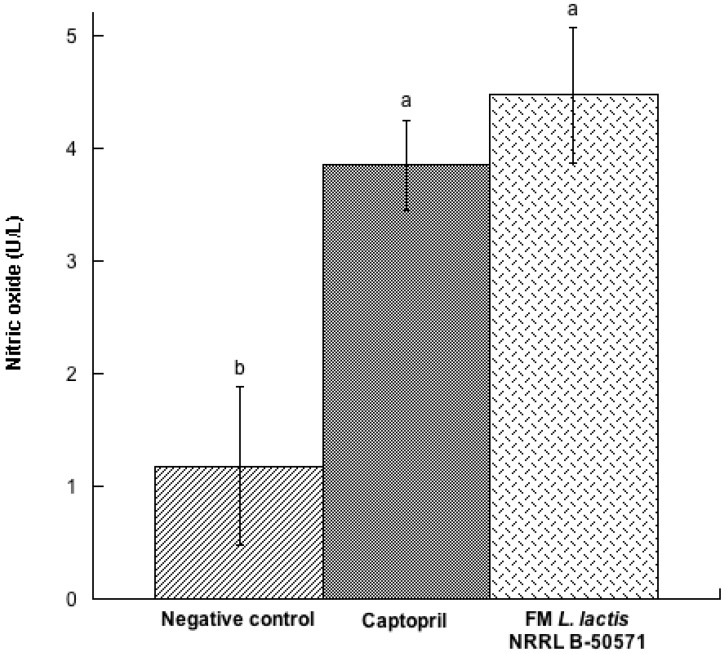
Nitric oxide (U/L) in plasma from spontaneously hypertensive rats after long-term treatments. Negative control: purified water; Captopril: 40 mg/kg body weight; FM *L. lactis* NRRL B-50571 (ad libitum): fermented milk with *Lactococcus lactis* NRRL B-50571. Data are presented as means ± SEM. Data sharing the same letter are not significantly different (*p* > 0.05).

In our present study, NO concentration in plasma was determined (Figure 6). After long-term effect of each treatment, plasma NO concentrations were significantly (*p* < 0.05) higher in the FM group and Captopril group than in the negative control group. Actually, plasma NO was 1.6 times higher in the FM group than in the negative control group; and was significantly different (*p* < 0.05). Recent studies have reported that some antihypertensive peptides may improve NO bioavailability through antioxidant effects, but certain peptides may also enhance NO production, improve endothelial function and improve blood pressure [31]. NO is an important bioregulatory molecule, which improves endothelial function, improves vasodilatation and controls blood pressure; therefore it is considered to be the main vasodilator. SHR may develop endothelial dysfunction by reducing bioavailability of NO. Therefore, increased bioavailability of NO may also improve vasodilation and reduce blood pressure [32,33]. Our findings are similar with those by Kim et al. [33]; they reported that fermented milk had an antihypertensive effect in SHR, had less ACEI activity in plasma, and more concentration of NO, compared to their control group. Hence, NO production may also be considered a mechanism involved in the hypotensive effect of FM with *L. lactis* NRRL B-50571.

### 3.5. Antioxidant Effect

To date, there is growing evidence that oxidative stress is one of the main responsible factors for the initiation or evolution of hypertension and its complications. Increased oxidative stress is also considered to be an important causative factor of the vascular endothelial dysfunction, causing decrement of NO production [34]. Therefore, there has been a rising interest on the pursuit for bioactive peptides with antioxidant activity, which may provide additional benefit to the endogenous antioxidant defense system [35]. Moreover, lipid peroxidation plays a major role in oxidative stress. Hence, bioactive components that may reduce lipoperoxidation may help decrease oxidative stress [34]. It has been reported that food bioactive peptides have strong antioxidant effect without significant side effects [36], and that milk derived peptides are the most studied [25]. In fact, it has been reported that whey protein has beneficial effects through enhancement of antioxidant enzymes and down regulation of oxidative markers such as lipoperoxidation [37]. 

**Figure 7:**
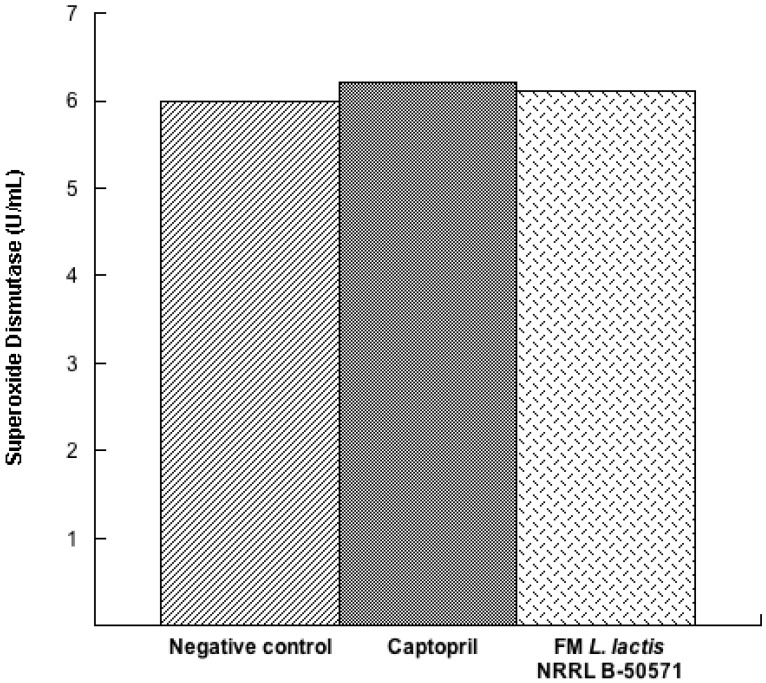
Superoxide dismutase (U/mL) in plasma from spontaneously hypertensive rats after long-term treatments. Negative control: purified water; Captopril: 40 mg/kg body weight; FM *L. lactis* NRRL B-50571 (ad libitum): fermented milk with *Lactococcus lactis* NRRL B-50571. Data are presented as median; and were analyzed by non-parametric test (Kruskal–Wallis *p* < 0.05).

**Figure 8:**
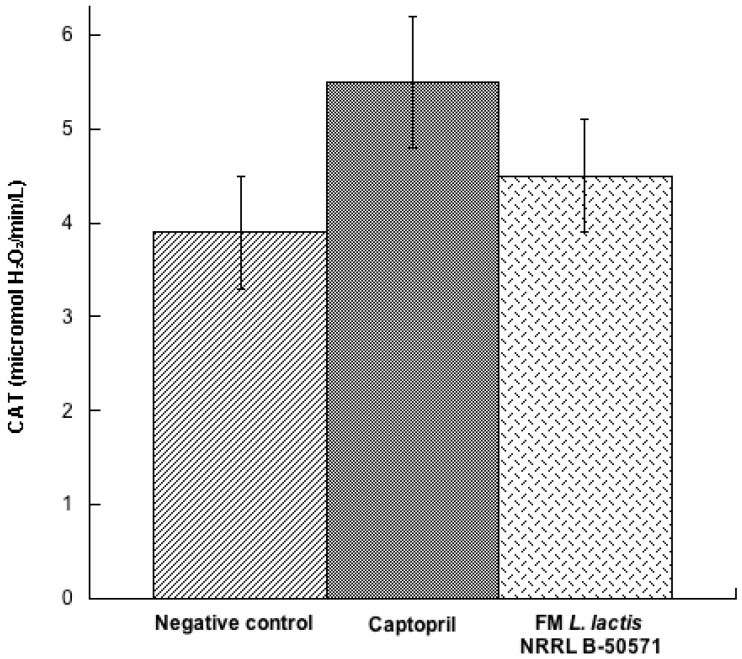
Catalase activity (CAT) (micromol H_2_O_2_/min/L) in plasma from spontaneously hypertensive rats after long-term treatments. Negative control: purified water; Captopril: 40 mg/kg body weight; FM *L. lactis* NRRL B-50571 (ad libitum): fermented milk with *L. lactis* NRRL B-50571. Data are presented as means ± SEM, and were analyzed by one-way ANOVA.

**Figure 9:**
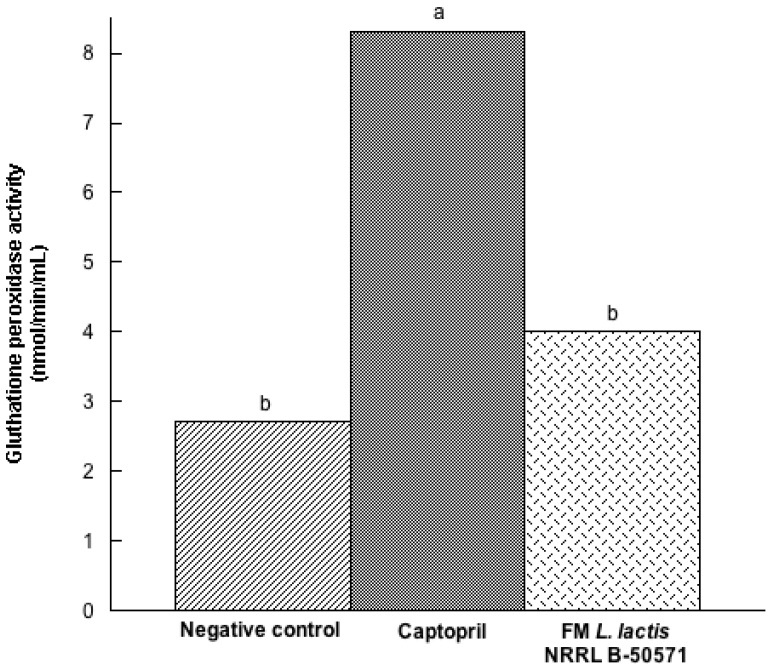
Glutathione peroxidase activity (nmol/min/mL) in plasma from spontaneously hypertensive rats after long-term treatments. Negative control: purified water; Captopril: 40 mg/kg body weight; FM *L. lactis* NRRL B-50571 (*ad libitum*): fermented milk with *Lactococcus lactis* NRRL B-50571. Data are presented as median; and was analyzed by non-parametric test (Kruskal–Wallis *p* <0.05). Data sharing the same letter are not significantly different (*p* > 0.05).

**Figure 10:**
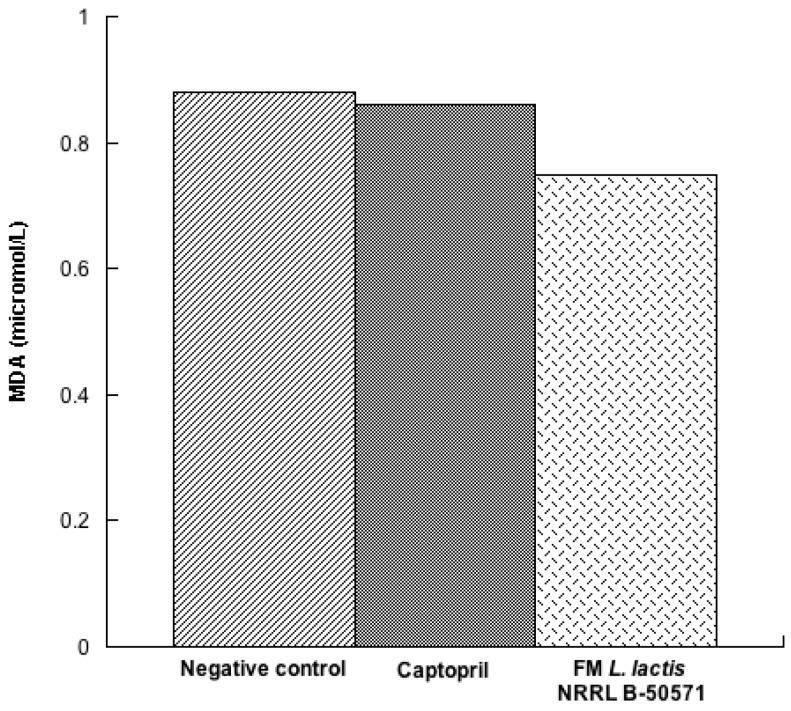
Lipid peroxidation represented as malondialdehyde (MDA) content in plasma from spontaneously hypertensive rats after long-term treatments. Negative control: purified water; Captopril: 40 mg/kg body weight; FM *L. lactis* NRRL B-50571 (ad libitum): fermented milk with *L. lactis* NRRL B-50571. Data are presented as median; and were analyzed by non-parametric test (Kruskal–Wallis *p* < 0.05).

In the present study, we evaluated the concentration of three antioxidant enzymes in plasma post-treatments. Results indicated that SOD (Figure 7) and CAT (Figure 8) were not significantly different (*p* > 0.05) in either group; nevertheless, values were higher in the Captopril group and the FM group than in the negative control group. Moreover, GPx activity (Figure 9) in the Captopril group was significantly higher (*p* < 0.05) than in the FM group and negative control group; however, GPx activity value from the FM group was slightly higher than in the negative control group. Additionally we evaluated lipoperoxidation in plasma through TBA, by detecting MDA products (Figure 10). In this study, although we did not detect differences between all groups (*p* > 0.05), MDA values from the FM group and Captopril group were lower than in the negative control. 

**Figure 11:**
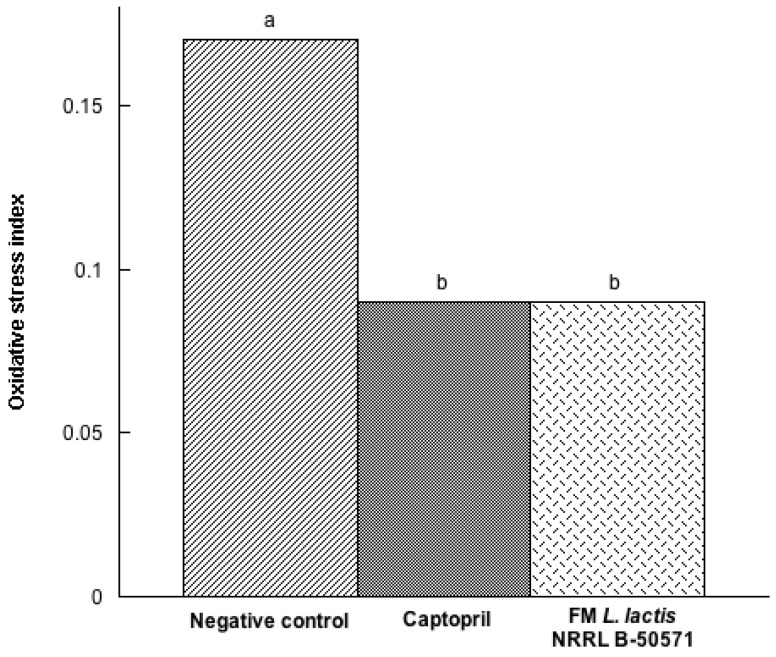
Oxidative stress index represented as the balance between lipid peroxidation (MDA) and antioxidant enzymes (Catalase and Glutathione peroxidase), from spontaneously hypertensive rats after long-term treatments. Negative control: purified water; Captopril: 40 mg/kg body weight; FM *L. lactis* NRRL B-50571 (ad libitum): fermented milk with *L. lactis* NRRL B-50571. Data are presented as median; and was analyzed by non-parametric test (Kruskal–Wallis *p* < 0.05). Data sharing the same letter was not significantly different (*p* > 0.05).

Nonetheless, after the evaluation of the oxidative stress index (Figure 11), which indicates the balance between lipoperoxidation (as malondialdehyde, MDA) and total antioxidant enzyme activity (CAT and GPx), results demonstrated that the Captopril group and the FM group were not significantly different (*p* > 0.05), yet they were significantly different (*p* < 0.05) from the negative control group. Since FM decreased oxidative stress in SHR, antioxidant activity may also be considered as an underlying mechanism pathway on the antihypertensive effect of FM. Thus, daily consumption of fermented milk with *L. lactis* NRRL B-50571 may help lower high blood pressure, as well as MDA levels and oxidative stress index, ACEI activity, and an enhancement of NO production. 

Similarly, it was reported that the antihypertensive effect of whey protein concentrate hydrolysates in SHR was through ACEI activity, oxidative damage reduction, and enhancement of NO production [38].

## 4. Conclusions

The antihypertensive effect of fermented milk with *Lactococcus lactis* NRRL B-50571 in SHR and prehypertensive subjects was previously reported. Nonetheless, this is the first study that elucidates the basic mechanistic pathways underlying the hypotensive effect of fermented milk with *Lactococcus lactis* NRRL B-50571. Results indicated that fermented milk with *Lactococcus lactis* NRRL B-50571 seem to act as angiotensin converting enzyme inhibitor, nitric oxide production enhancer and as an antioxidant; which overall helped reduce blood pressure in SHR. 

## Figures and Tables

**Table 1:** Clinical characteristics of SHR.

Groups	Negative Control (Purified Water)	WSE-FM NRRL B-50571	Naloxone + WSE-FM NRRL B-50571	*p* Value
Weight	319 ± 20.1	321.9 ± 16	322.6 ± 10.24	0.97
SBP (mmHg)	188.1 ± 21.8	186.3 ± 20	189 ± 8.8	0.99
DBP (mmHg)	126.2 ± 24.3	127 ± 13.2	131.6 ± 10.7	0.83
Heart rate (beats/min)	488.8 ± 35.1	465.2 ± 27.7	453.7 ± 28.9	0.13

SHR: spontaneously hypertensive rats; SBP: systolic blood pressure; DBP: diastolic blood pressure; WSE: water soluble extract; FM: fermented milk.

**Table 2:** Clinical characteristics of SHR.

Groups	Negative Control (Purified Water)	Captopril	FM NRRL B-50571	*p* Value
Weight	341.5 ± 18.5	357.2 ± 12.3	340.5 ± 17.9	0.70
SBP (mmHg)	200.8 ± 16.1	201.8 ± 18.3	201.8 ± 13.1	0.98
DBP (mmHg)	152 ± 23.3	158 ± 30.2	150.1 ± 21.8	0.78
Heart rate (beats/min)	461.7 ± 33.5	442.6 ± 40.1	433.7 ± 38	0.28

SHR: spontaneously hypertensive rats; SBP: systolic blood pressure; DBP: diastolic blood pressure; FM: fermented milk.
